# Small Incision Combined with Nephroscope Operation in the Treatment of Infectious Pancreatic Necrosis: A Single-Center Experience of 37 Patients

**DOI:** 10.1155/2021/9910058

**Published:** 2021-05-13

**Authors:** Yinghui Song, Guoguang Li, Hongwei Zhu, Zhangtao Yu, Bo Jiang, Chuang Peng, Sulai Liu

**Affiliations:** ^1^Department of Hepatobiliary Surgery/Hunan Research Center of Biliary Disease, Hunan Provincial People's Hospital/The First Affiliated Hospital of Hunan Normal University, Changsha, 410005 Hunan Province, China; ^2^Biliary Disease Research Laboratory of Hunan Provincial People's Hospital, Key Laboratory of Hunan Normal University, Changsha, 410005 Hunan Province, China; ^3^Clinical Medical Technology Research Center of Hunan Provincial for Biliary Disease Prevention and Treatment, Changsha, 410005 Hunan Province, China; ^4^Department of Hepatobiliary and Pancreatic Surgery, The Third Xiangya Hospital, Central South University, Changsha, 410018 Hunan Province, Hunan, China

## Abstract

**Objective:**

To explore the safety and efficacy of small incision combined with nephroscope surgery in the treatment of infectious pancreatic necrosis.

**Methods:**

A retrospective analysis of the clinical data of 37 patients with infectious pancreatic necrosis who underwent small incision combined with nephroscopy in the Department of Hepatobiliary Surgery of Hunan Provincial People's Hospital from January 2018 to December 2019.

**Results:**

All 37 patients successfully completed small incision combined nephroscope surgery. The median time from the onset to the operation of all patients was 38 days (range: 29-80 days), and the hospital stay was 19 days (range: 3-95 days). The median number of drainage tubes placed during the operation was 4 (range: 2-8). According to the different surgical approaches, 13 cases were through the retroperitoneal approach, 11 cases were through the omental sac approach, 2 cases were through the intercostal approach, and 11 cases were combined approach. The operation time was 85.3 ± 31.6 min, and intraoperative bleeding was 63.1 ± 40.0 ml. The incidence of complications (Clavien-Dindo grade 3 and above) was 5.4%. Among them, 2 patients were admitted to the intensive care unit due to postoperative bleeding, 1 case was cured by conservative treatment, and 1 case was cured by interventional treatment. During the follow-up period, 2 patients developed colonic fistula at 2 weeks after operation, and 2 patients developed gastric fistula at 1 week and 3 weeks after operation; all were cured by conservative treatment.

**Conclusion:**

Small incision combined with nephroscope surgery is an effective treatment for patients with infectious pancreatic necrosis by removing necrotic tissue, unobstructed drainage, and reducing complications.

## 1. Introduction

Acute pancreatitis (AP) is a complex inflammatory disease, which can be divided into interstitial edema pancreatitis and necrotizing pancreatitis according to the pathological type. Necrotizing pancreatitis is characterized by the necrosis of the pancreatic parenchyma and/or peripancreatic tissue, which is the pathophysiological basis for the progression of moderate to severe acute pancreatitis [1]. Infected pancreatic necrosis (IPN) is one of the complications of severe acute pancreatitis (SAP), which can further develop blooding, thrombosis, gastrointestinal fistula, intestinal obstruction, sepsis, etc., and its mortality can be as high as 30-39% [2]. Surgeons play an important role in the comprehensive treatment of acute pancreatitis. For patients with IPN, percutaneous catheter drainage (PCD) may benefit for most patients. Effective surgery intervention is needed when drainage is ineffective [3, 4]. The author retrospectively analyzed the clinical data of 37 patients with infectious pancreatic necrosis who underwent small incision combined with nephroscopy in the Department of Hepatobiliary Surgery of Hunan Provincial People's Hospital from January 2018 to December 2019 to provide new treatment options for patients with infectious pancreatic necrosis.

## 2. Materials and Methods

### 2.1. General Information

A retrospective analysis of the clinical data of 37 patients with IPN who underwent small incision combined with nephroscopy in the Department of Hepatobiliary Surgery of Hunan Provincial People's Hospital from January 2018 to December 2019, including 24 males and 13 females was performed; the median age was 50 (16-68) years old. All patients had no family history, 11 had biliary stones, 10 had hyperlipidemia, and 1 had a history of alcohol abuse. All patients underwent small incision combined nephroscope operation successfully. This study was approved by the medical ethics committee of the hospital, and all patients signed an informed consent form.

### 2.2. The Diagnosis of IPN

When infectious pancreatic necrosis is suspected clinically, the diagnosis can be confirmed if one of the following two is met: (1) Fine needle aspiration or PCD drainage is positive for bacterial or fungal culture. (2) CT examination of the image showed air bubbles in the pancreas and peripancreatic necrosis area [5].

### 2.3. Surgical Methods

#### 2.3.1. Preoperative Preparation

All cases taken whole abdomen scan + enhanced CT to determine the location and size of infectious pancreatic necrosis, and the relationship with the surrounding organs before the operation. A projection of the abscess was drawn on the surface of each patient. Some suitable patients were treated with PCD.

#### 2.3.2. Surgical Approach

*(1) Transretroperitoneal Approach Surgery*. After general anesthesia, the patients were placed at lateral position. The surgeon took an oblique incision about 3-6 cm in length according to the preoperative ultrasound positioning and enter the posterior peritoneum layer by layer. Then, toothless oval forceps were used to clamp out the necrotic tissue gently. For the remaining necrotic tissue, a pulse water gun was applied to rinse the tissue at low pressure. If there are remaining residuals, nephroscope clamps combined with flushing were used to remove the necrotic tissue as cleanly as possible. At last, multiple drainage tubes were retained.

*(2) Transomental Sac Approach Surgery*. After general anesthesia, the surgeon generally took a median incision or a longitudinal incision at the preoperative PCD puncture point, which is about 3-6 cm in length and then entered the abdominal cavity layer by layer. Next, gauze/saline was used to pad to surround the incision for a circle to prevent the spread of pus. Then, the surgeon opened the gastrocolic ligament into the abscess cavity in the omental sac bluntly. After suctioning the pus, toothless oval forceps, pulse water gun to rinse, and combined nephroscope were used to remove necrosis as thoroughly. At last, multiple drainage tubes were retained.

*(3) Transcostal Approach Surgery*. After general anesthesia, the patients were placed at lateral position to expand the intercostal space as much as possible. The surgeon took the left side incision in the intercostal space closest to the abscess, or the preoperative PCD puncture point incision, about 3 cm in length, entered the abscess cavity layer by layer, sucked up the pus, used toothless oval forceps, pulse water gun to rinse, and combined nephroscope to remove necrosis and pus as thoroughly as possible, and retained multiple drainage tubes.

#### 2.3.3. Postoperative Treatment

In view of the possibility that there may be a small amount of necrotic tissue or pus, the patient should be flushed with normal saline at low pressure or repeatedly lavage with a syringe to remove the remaining necrotic tissue or pus for at least three days.

### 2.4. Follow-Up

The patients' gender, age, symptoms, the complete recovery time, and long-term complications after operation were followed up in outpatient and telephone visits after the operation. The follow-up time was until February 1, 2020.

### 2.5. Statistical Methods

SPSS19.0 software was applied for data analysis. CRP, WBC, and PCT were analyzed by *t*-test. *p* < 0.05 was considered statistically significant.

## 3. Results

### 3.1. Surgery and Postoperative Situation

**Figure 1:**
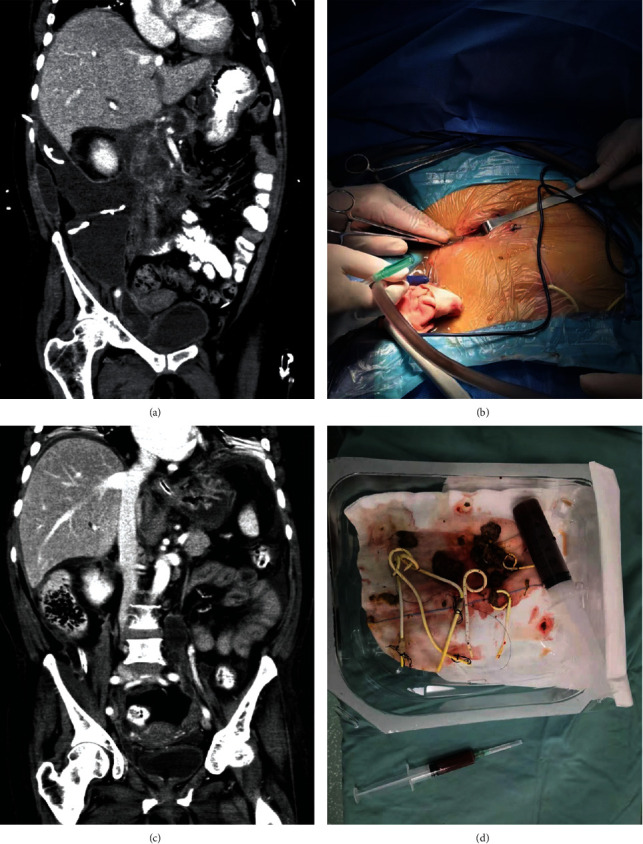
Small incision combined with nephroscope surgery for infectious pancreatic necrosis. (a) Preoperative CT showed a large amount of necrosis in the pancreas and intestinal space. (b) Incision of the transretroperitoneal approach surgery. (c) Postoperative CT showed that the necrosis was basically removed and absorbed. (d) Necrosis and exudates removed.

All 37 patients successfully completed small incision combined nephroscope operation (Figure 1). The median time from the onset to the first operation of all patients was 38 days (range: 29-80 days); the median number of drainage tubes placed on average was 4 (range: 2-8). According to different surgical approaches, 13 cases were through the retroperitoneal approach, 11 cases were through the omental sac approach, 2 cases were through the intercostal approach, and 11 cases were combined approach. The operation time was 85.3 ± 31.6 min, and intraoperative bleeding was 63.1 ± 40.0 ml. At 72 h after operation, postoperative c-reactive protein (CRP), white blood cell count (WBC), and procalcitonin (PCT) decreased significantly. The average Acute Physiology and Chronic Health Evaluation II(APACHE II) score was 15.68 ± 3.22, and the *R* value was 20.15 ± 13.46 (%), see Table 1 for details.

The average postoperative hospital stay of all patients was 19 (3-95) days. The average duration of antibiotic use after surgery was 8 (5-21) days. There was one case that underwent reopen surgery to remove infectious necrosis 15 days after operation because of poor curative effect and persistent high fever. The incidence of perioperative Clavien-Dindo grade 3 and above complications was 5.4% (2/37). One case was treated with interventional splenic artery embolization to stop bleeding due to splenic artery rupture and hemorrhage and returned to the general ward 3 days later. Another case gone blooding 4 days after operation and then was sent to the intensive care unit for conservative treatment. After 3 days of nonsurgical hemostasis treatment, he returned to the general ward. There were no perioperative deaths.

### 3.2. Microbial Culture Results

The results of microbial culture of pus showed that the infectious pathogens were Klebsiella pneumoniae (12 cases), Acinetobacter baumannii (8 cases), Escherichia coli (3 cases), Enterobacter cloacae (3 cases), Enterococcus faecium (2 cases), and Staphylococcus aureus (2 cases). And there were 7 cases that have mixed infections caused by two or more pathogenic bacteria, of which 3 cases were combined with fungal infections.

### 3.3. Follow-Up

The follow-up period of all patients was 3 to 24 weeks. Two cases developed colonic fistula 2 weeks after operation, and 2 cases developed gastric fistula 1 week and 3 weeks after operation, and they were cured by conservative treatment. All patients had varying degrees of malnutrition, and all improved after nutritional support intervention. There were no new cases of diabetes or pancreatic dyspepsia.

## 4. Discussion

The comprehensive treatment model of acute pancreatitis based on multidisciplinary team diagnosis and treatment can reduce the incidence of death due to reducing early systemic inflammatory response syndrome and multiple organ failure. The number of patients who died of shock and multiple organ failure is still high for IPN causing severe sepsis and secondary infection in the later stage of the disease [6, 7].

Traditional surgical intervention mainly refers to removal of the pancreatic necrotic tissue by open abdominal cavity (operate debridement (OPD)). Beger et al. performed open debridement for pancreatic necrotic tissue removal and pointed that it was very important including restricted necrosectomy and postoperative local lavage treatment for IPN [8]. Subsequently, another study pointed out that the direct retroperitoneal approach could remove the necrosis by several reoperations without the risk of large wound dehiscence [9]. However, traditional laparotomy has many complications including abdominal bleeding, gastrointestinal fistula and others causing by interfering to abdominal organs, large trauma, and long drainage path. Minimally invasive necrosectomy seems superior to open necrosectomy in terms of complications [10]. In another study comparing video-assisted retroperitoneal debridement (VARD) and small incision pancreatic necrosectomy (SIPN) for IPN, SIPN may be superior to the step-up approach for patients with IPN than for those with VARD because SIPN has the advantages of shorter hospital stay, lower total cost, and lower reintervention rate [11]. Currently, many centers are implementing minimally invasive endoscopic step-up approaches for the treatment of IPN [12]. We adopt a small incision combined with nephroscope surgery to reach the abscess cavity for debridement. This surgical method was based on the minimal access retroperitoneal pancreatic necrosectomy (MARPN) surgical technique, which has a small impact on the patient, and can remove a large amount of necrotic tissue and quickly alleviate the symptoms of systemic poisoning in the patient.

Percutaneous catheter drainage (PCD) is the preferred method of trauma escalation strategy [13]. However, surgical intervention is required when PCD alone cannot effectively control the disease by persistent organ failure, increased proportion of pancreatic necrosis, mixed lesions of cysts and solids, mixed infection, etc. In this study, the patients who experienced PCD had poor efficacy or did not have the conditions to perform PCD. Furthermore, all of them had duration of IPN more than 4 weeks.

At present, there are many reports on minimally invasive treatment of IPN [14]. A retrospective analysis of 50 SAP cases that underwent laparoscopic surgery showed the complication rate was 38% and the mortality was 10% [15]. However, laparoscopic surgery has the risk of spreading retroperitoneal infections into the peritoneal cavity during the operation of the abdominal cavity, and the establishment of pneumoperitoneum may affect the stability of circulation in severe patients. In contrast, small incision combined with nephroscope surgery could directly enter the abscess cavity to avoid side damage to the abdominal organs. Also, the drainage tube does not pass through the abdominal cavity to reduce the incidence of abdominal cavity infection. At the same time, drainage tube does not compress the digestive tract to reduce the occurrence of a gastrointestinal fistula. In this study, there were 4 in 37 patients that developed intestinal fistula and gastric fistula. The incidence of gastrointestinal fistula is lower than laparoscopic surgery.

Another systematic review retrospectively analyzed 455 patients who underwent endoscopic pancreatic necrosis removal surgery. Each patient performed the procedure 4 times on average [16]. 81% of the patients were cured by endoscopy. The complications incidence rate was 36%, and the fatality rate was 6%. However, it is mainly suitable for the removal of necrotic foci of infection close to the stomach or duodenum. At the same time, the thoroughness of the removal is not as good as small incisions. In this study, blunt finger separation was used to avoid sharp damage to the surrounding vital organs and blood vessels, reducing the risk of bleeding. There were 2 patients that had postoperative bleeding. One case was treated with interventional splenic artery embolization to stop the bleeding due to rupture of the splenic artery and returned to the general ward after 3 days. Another one was transferred into the intensive care unit 4 days after the operation, and the bleeding was successfully stopped by conservative treatment for 3 days.

The implementation of small incision combined with nephroscope surgery in the treatment of infectious pancreatic necrosis requires the following prerequisites: (1) preoperative accurate reading of CT and other images, combined with the PCD drainage fluid bacterial culture results, to confirm the diagnosis of IPN; (2) determine the location of the infection and necrosis that is close to the body surface, determine the degree of liquefaction of the necrosis, and determine that the patient is suitable for minimally invasive surgery; and (3) try to postpone the operation time until 4 weeks after the onset of the disease. At this time, the boundary between the necrotic lesion and the normal tissue is clear, which helps to preserve more normal tissue, which is beneficial to the recovery of pancreatic internal and exocrine function and reduces postoperative complications. In summary, the characteristics of the patients in the study found that the average APACHE II was 15.68 ± 3.22. It is well known that the APACHE II score had statistical importance and prediction of outcome in AP, forecasting patients at risk of SAP [17]. All patients in this study were relatively young, only some patients with diabetes and no other serious comorbidities, the compensation of each organ function is better, so the effect is better. Also, it is reported that the level of CRP is related to the severity of acute pancreatitis. In this study, the average was 63.47 ± 46.07 mg/l, which was lower than that reported in other studies [18, 19]. It is indicated that the patients in this study had less severe inflammation. At 72 h after operation, all of the CRP, WBC, and PCT were lower than before operation (*p* < 0.05). It is suggested that after removal the pancreatic necrotic tissue by small incision combined with nephroscope operation could relieve inflammation effectively.

In this study, there was one case that underwent reopen surgery to remove infectious necrosis 15 days after operation because of poor curative effect and persistent high fever. The reason for the reoperation was considered: the patient's abscess was scattered, especially the necrotic tissue located at the root of the mesentery was not completely removed during the initial operation. It may be solved with soft endoscopy during or after operation, but more experience is needed.

In summary, the patients with relatively concentrated infectious pancreatic necrosis and no obvious gastrointestinal fistula were suitable for small incision combined with nephroscope to remove infectious pancreatic necrosis. It combined with intraoperative pulsed water gun flushing had the advantages of fast speed, thorough debridement, exact curative effect, and less trauma. Surgical incision approach could be selected according to the distribution of infectious necrotic tissue through the omental sac, retroperitoneal approach, or intercostal approach. This surgical method combines the advantages of puncture, endoscopic, and open surgery. Also, it is simple and easy to implement. Moreover, it can also be promoted in primary hospitals.

## Figures and Tables

**Table 1:** Clinical data of the 37 patients with infected necrotizing pancreatitis.

Clinical data	IPN (*N* = 37)	
Age (years)	49.41 ± 10.70	
Gender	Male	24
	Female	13
Cause of disease [n (%)]	Hyperlipemia	14 (37.84%)
	Cholelithiasis	17 (45.95%)
	Alcoholism	6 (16.21%)
Surgical approach	Transretroperitoneal approach surgery	13 (35.14%)
	Transomental sac approach surgery	11 (29.73%)
	Transcostal approach surgery	2 (5.40%)
	Combined approach surgery	11 (29.73%)
APACHE II score before operation	15.68 ± 3.22	
*R* value(%)	20.15 ± 13.46	
Bleeding volume (ml)	66.35 ± 55.89	
C reactive protein (mg/l)	Before operation	63.47 ± 46.07
	At 72 h after operation	37.03 ± 33.86^∗^
White blood cell count (^∗^10^9^/l)	Before operation	11.63 ± 6.72
	At 72 h after operation	8.21 ± 3.68^∗^
Procalcitonin (ng/ml)	Before operation	1.88 ± 1.65
	At 72 h after operation	0.38 ± 0.21^∗^

^∗^Compared with that before operation, *p* < 0.05.

## Data Availability

Data and materials are included in the manuscript.
